# A single-institution retrospective study of multicentric gliomas stratified by *IDH* mutational status

**DOI:** 10.1093/noajnl/vdag047

**Published:** 2026-05-10

**Authors:** Chuyin Yang, Ryan Mostafavi, Collin Le, Addison Fisher, Blaine S C Eldred, Terry J Prins, Thomas J Lai, Linda M Liau, Richard G Everson, Robert A Chong, Phioanh L Nghiemphu, Timothy F Cloughesy, Benjamin M Ellingson, Albert Lai

**Affiliations:** Department of Neurology, University of California, Los Angeles, Los Angeles, California, USA; Department of Neurology, University of California, Los Angeles, Los Angeles, California, USA; Department of Neurology, University of California, Los Angeles, Los Angeles, California, USA; Department of Neurology, University of California, Los Angeles, Los Angeles, California, USA; Department of Neurology, University of California, Los Angeles, Los Angeles, California, USA; Department of Neurology, University of California, Los Angeles, Los Angeles, California, USA; Department of Neurosurgery, University of California, Los Angeles, Los Angeles, California, USA; Department of Neurosurgery, University of California, Los Angeles, Los Angeles, California, USA; Department of Neurosurgery, University of California, Los Angeles, Los Angeles, California, USA; Department of Neurology, University of California, Los Angeles, Los Angeles, California, USA; Department of Neurology, University of California, Los Angeles, Los Angeles, California, USA; Department of Neurology, University of California, Los Angeles, Los Angeles, California, USA; Department of Radiology, University of California, Los Angeles, Los Angeles, California, USA; Department of Neurology, University of California, Los Angeles, Los Angeles, California, USA

**Keywords:** glioma, *IDH1/2*, metachronous, multicentric, synchronous

## Abstract

**Background:**

Multicentric glioma (MCG) are defined radiographically as two or more tumor foci with separation of MRI T2-FLAIR (T2-weighted Fluid-attenuated Inversion Recovery) hyperintensities presenting synchronously (sMCG) and/or metachronously (mMCG). Previous studies have not stratified *isocitrate dehydrogenase* (*IDH*) wild-type and mutant gliomas by sMCG and mMCG. In this large, single-institution cohort stratified by *IDH* status, we evaluated MCG prevalence, prognostic implications, time to mMCG (TtM), location, and pathological concordance.

**Methods:**

We identified 836 *IDH* wild-type and 531 *IDH* mutant diffuse glioma patients treated at our institution with evaluable MRI. We inspected MRIs at presentation for sMCG and subsequent imaging for mMCG, reviewed radiology and pathology reports, and performed overall survival (OS) and TtM analyses.

**Results:**

MCG prevalence was higher in *IDH* wild-type cases (18%) than in *IDH* mutant cases (9%) (*P*  <  .0001). We found 20 sMCG and 26 mMCG in *IDH* mutant and 91 sMCG and 54 mMCG in *IDH* wild-type patients. In 7 wild-type and 1 mutant instances, mMCG arose from sMCG (smMCG). While sMCG and mMCG were associated with lower OS in *IDH* wild-type cases (sMCG: HR  =  1.46, *P*  =  .003; mMCG: HR  =  1.44, *P*  =  .02), only mMCG was associated with lower OS in *IDH* mutant cases (sMCG: HR  =  1.22, *P*  =  .7; mMCG: HR  =  2.64, *P*  =  .001). *IDH* wild-type cases had shorter TtM than mutant cases (*P*  <  .0001). Among double-biopsied MCG, pathology discordance occurred in 5/7 mutant but 0/28 wild-type cases.

**Conclusion:**

Results from this cohort of diffuse gliomas stratified by *IDH* status showed differences between *IDH* wild-type and mutant cohorts in prevalence, prognosis, TtM, distribution, and pathological concordance.

Key Points

- The prevalence of MCG is higher in *IDH* wild-type patients than in *IDH* mutant patients.
- In *IDH* mutant patients, mMCG but not sMCG are associated with lower OS than non-MCG.
- *IDH* mutant but not wild-type MCG had discordant pathologies between tumor foci.

Importance of the Study

Multicentric glioma (MCG) can present synchronously (sMCG) and/or metachronously (mMCG). We expand on current understanding of MCG in this retrospective study of 836 *IDH* wild-type and 531 *IDH* mutant glioma patients. MCG has higher prevalence in *IDH* wild-type (18%) than mutant gliomas (9%). While both sMCG and mMCG are associated with lower OS in *IDH* wild-type cases, only mMCG are associated with lower OS in *IDH* mutant cases. TtM is longer in the *IDH* mutant than in the wild-type cohort. Amongst both *IDH* mutant and wild-type MCG, mMCG demonstrated greater infratentorial involvement than sMCG. Among double-biopsied MCG, we observed pathology discordance between lesions in 5/7 of *IDH* mutant and none of *IDH* wild-type patients, suggesting that multiple biopsies should be considered for *IDH* mutant MCG when clinically feasible and since discordance could alter management decisions. These results demonstrate key differences between *IDH* mutant and wild-type MCG.

Gliomas typically present as a single tumor focus with variable levels of T2-weighted fluid-attenuated inversion recovery (T2-FLAIR) hyperintensity.^1^ However, gliomas with two or more foci have also been documented previously in the literature. Multicentric gliomas (MCG) are a radiographically defined subgroup of glioma as the occurrence of two or more independent tumor foci,^2-4^ which can occur synchronously (sMCG) and/or metachronously (mMCG).^3^^,^^5^^,^^6^ MCG is distinct from multifocal glioma by requiring the lack of continuous T2-FLAIR hyperintensity connecting the foci on MRI.^2-4^ Additionally, while multifocal glioblastoma is thought to be monoclonal in origin,^7^ multicentric glioblastoma is thought to have independent origin for each tumor focus.^8^ MCG are therefore more likely than multifocal glioma to be biologically distinct from non-MCG. Meta-analyses of MCG have determined incidence rates of 1%-35% in glioblastoma^3^^,^^9^ and 2%-10% in low grade gliomas.^6^ Furthermore, while previous studies have correlated MCG with poorer prognostic outcomes in glioblastoma,^10^^,^^11^ literature examining the effects of MCG on *isocitrate dehydrogenase* (*IDH*) mutant and low-grade gliomas has proven to be inconclusive due to inadequate sample size.^6^^,^^12^

*IDH* mutation status has emerged as a key genetic characteristic that has informed the clinical management and prognosis of glioma patients.^13-15^ Although MCG in *IDH* wild-type glioblastoma have been better studied, the prognosis and characteristics of *IDH* mutant MCG remain poorly understood. Additional studies on the clinical performance of MCG patients compared to their non-MCG counterparts have also been largely restricted to exclusively *IDH* wild-type or mutant cohorts, and there have not been large cohort studies directly comparing both *IDH* mutant and wild-type MCG in the same cohort. Given the poor prognosis of MCG in *IDH* wild-type glioblastoma, the lack of studies on MCG in *IDH* mutant gliomas, and poor characterization of synchronous and metachronous MCG, we analyzed 836 *IDH* wild-type and 531 *IDH* mutant diffuse glioma patients treated at UCLA with evaluable MRI imaging. We characterized mMCG and sMCG subtypes in the context of *IDH* mutational status, compared *IDH* wild-type and mutant MCG, and examined the concordance of pathology between the MCG lesions within each patient.

## Methods

### Patient Cohort

In this institutional board-approved study, in which informed patient consent was obtained prior to the collection of patient data, we retrospectively identified a cohort of 836 *IDH* wild-type and 531 *IDH* mutant patients with diffuse glioma treated at UCLA. During the derivation of the cohort, we expanded the time interval of inclusion for *IDH* mutant patients to increase the cohort size of mutant MCG. We identified consecutive *IDH* wild-type glioblastoma and gliosarcoma patients who presented to UCLA from 2013 to 2023 and *IDH* mutant patients from 2007 to 2025. For inclusion into this patient cohort, we required a known *IDH* mutational status, imaging prior to or within three months after their initial surgery, and diagnosis of diffuse glioma (see “Pathological Analysis” below). While all patients were ≥18 years of age when initially seen at UCLA, seven patients were diagnosed when they were <18.

### Imaging Identification of MCG, mMCG, and sMCG

For each patient, we analyzed corresponding T2-FLAIR and T1-weighted contrast enhanced MRI sequences. We defined MCG to be ≥2 tumors with non-overlapping T2-FLAIR hyperintensities.^1-4^^,^^16-18^ As such, the imaging criteria of MCG are based solely on separation of T2-FLAIR hyperintense lesions irrespective of the number of distinct T1-weighted contrast enhanced lesions. Non-multicentric gliomas include those with a single lesion and those with multiple apparent lesions connected via T2-FLAIR hyperintensity. According to previous studies,^1-4^^,^^16^^,^^17^ the latter are considered multifocal, not MCG, and were categorized as non-MCG cases in our analyses. We further subset our cohort into sMCG and mMCG (Supplementary Figure S1). In sMCG, the patient presented with MCG *de novo* in their initial MRI study.^3^ Gliomas were considered mMCG if they were initially non-MCG but later developed an additional, independent lesion after treatment that was remote from the initial tumor.^3^^,^^18^ As some patients lacked available MRI prior to initial surgery, any patient who demonstrated MCG on an MRI obtained within three months of initial surgery was classified as sMCG, regardless of whether the additional lesion was present at diagnosis or appeared during this early interval. Patients without an MRI within this time frame were excluded from this cohort. Patients who acquired MCG before receiving initial surgery were included as sMCG. Evaluation for mMCG was performed using all subsequent surveillance MRIs beyond this three-month window. In cases where the T2-FLAIR hyperintensity of a new lesion overlapped with that of the resected original tumor, the new lesion was considered not discrete and therefore not MCG. Some patients acquired mMCG from sMCG and were considered smMCG.

TtM was defined as the time between initial diagnosis and the MRI scan demonstrating acquired mMCG. Because some patients had relatively short imaging follow-up, we also evaluated the time interval from the first to the last available MRI. Patients who did not develop mMCG during this time interval were censored for TtM. The median MRI follow-up duration was 51.7 months for *IDH* mutant patients and 11.2 months for *IDH* wild-type patients.

To determine whether additional foci of hyperintensity was glioma versus a nonspecific hyperintensity, the lesion was required to meet at least one of the following criteria: (1) the lesion was pathologically confirmed as a glioma, (2) the lesion was suspected as a neoplasm per radiology report, or (3) the lesion increased in size over time. Cases of gliomatosis were not considered to be MCG. Cases where distinct foci arose in patients with leptomeningeal spread were categorized as MCG.

### Pathological Analysis

Pathological diagnosis and molecular analysis were obtained from available pathology reports and additional clinical molecular testing. In 15 instances, *IDH* mutant patients were seen before the adoption of *IDH* mutation testing, and Sanger sequencing was used to determine *IDH* status retrospectively.^14^ For patients diagnosed prior to 2021, we applied 2021 WHO guidelines for the classification of tumor pathologies when adequate molecular data was available to update obsolete pathologies such as *IDH* mutated glioblastoma, mixed gliomas, and *IDH* wild-type astrocytoma (Supplementary Table S1). For *IDH* mutant patients, the presence of *1p19q* codeletion by fluorescence in situ hybridization (FISH) or next generation sequencing (NGS) was used to differentiate astrocytoma and oligodendroglioma. For inclusion by WHO 2021 guidelines (Group 1), oligodendroglioma patients must have *1p19q* codeletion, and astrocytoma patients must be negative for *1p19q* codeletion and have *CDKN2A/B* molecular data. In group 1, six grade 2 and 12 grade 3 astrocytomas were upgraded to grade 4 astrocytoma based on *CDKN2A/B* loss by FISH or NGS. Five patients with both FISH and NGS testing had discrepancies in results between the two methods; namely, all five had homozygous loss by FISH but retained *CDKN2A/B* by NGS. In these cases, we prioritized the FISH result and upgraded the patient’s diagnosis.

However, not all *IDH* mutant patients had molecular data available for *1p19q* codeletion and/or *CDKN2A/B* loss data, and as the WHO 2021 guidelines were utilized only when data was available, the guidelines were not uniformly applied to the cohort (Supplementary Table S1). 89 grade 2, 3, and 4 astrocytoma patients who lacked *CDKN2A/B* loss data but were negative for *1p19q* codeletion were included based on their WHO 2016 diagnosis (Group 2). For the 81 patients who lacked *1p19q* codeletion data, 63 diagnoses were inferred based on available *ATRX*, *TERT*, *TP53*, *CIC,* and *FUBP1* mutational statuses (Group 3). We further distinguished group 3 as inferred by WHO 2021 or 2016. All oligodendrogliomas were considered inferred WHO 2021; for astrocytoma pathologies, the presence or absence of *CDKN2A/B* data determined if the patient was categorized by WHO 2021 or 2016, respectively. 18 of the 81 lacked these molecular data and were included according to their initial diagnoses, which were based on histology only (Group 4). For *IDH* wild-type patients, we applied the 2021 guidelines to upgrade previous *IDH* wild-type astrocytomas into glioblastoma when sufficient molecular information was available. If there was not sufficient molecular data to upgrade legacy diagnoses according to the 2021 WHO guidelines for diffuse *IDH* wild-type glioma, we excluded these patients, who were possible molecular glioblastomas, from the cohort.

### Statistics

We utilized Kaplan-Meier (KM) and multivariate Cox regression for both OS and TtM analyses. We defined OS as the overall survival of the patient from the first surgery/pathological diagnosis to their censor date or death. TtM was defined as the time from the patient’s earliest available MRI study, either immediately before surgery or within three months after first surgery, to the date at which the metachronous lesion first appeared on T2-FLAIR imaging. For the KM analysis, the event is the acquisition of mMCG, and the patient was censored if they did not acquire mMCG within the time between their first and last available MRI.

To assess the effects of other factors on OS and TtM, we used Cox multivariate analysis with the following covariates: Karnofsky Performance Scale (KPS) after initial surgery, sex, age at diagnosis, *MGMT* methylation, extent of resection, whether the patient received temozolomide (TMZ), whether the patient received radiation therapy (RT), and pathology for analyses of *IDH* mutant patients. MCG status stratified and not stratified by sMCG and mMCG were also used for OS analysis. As categorical variables were largely segregated by *IDH* status, we also analyzed aggregate *IDH* mutant and wild-type data to evaluate interaction effects between MCG and *IDH* mutational status on OS using multivariate Cox regression. For categorical data, we used Fisher’s exact test and Chi-square. To compare cohort ages, we used Welch’s *t*-test. All statistical analyses were carried out using GraphPad Prism, and *P* value at or below .05 were considered statistically significant.

## Results

### Cohort Characteristics and Prevalences of Types of Multicentricity

From our initial database query for *IDH* wild-type glioma from 2013 to 2023 and *IDH* mutant glioma from 2007 to 2024, we identified 1367 total glioma patients. Of these, 836 were *IDH* wild type and 531 were *IDH* mutant. Relevant clinical characteristics, treatment, and molecular data were collected from available electronic health records (Table 1). As expected,^19^ the median age of patients in the *IDH* wild-type cohort was higher than those in the *IDH* mutant cohort (WT: 61 years, Mut: 37 years, *P*  <  .0001, Welch’s *t*-test), and both *IDH* wild-type and mutant cohorts had a higher proportion of males to females (WT: 520/836, 62%; Mut: 317/531, 60%). Of the included *IDH* wild-type pathologies, 828/836 (99%) were glioblastoma and 8/836 (1%) were gliosarcoma. Within *IDH* mutant pathologies, we identified 142/531 (27%) grade 2 oligodendroglioma (G2O), 68/531 (13%) grade 3 oligodendroglioma (G3O), 121/531 (23%) grade 2 astrocytoma (G2A), 112/531 (21%) grade 3 astrocytoma (G3A), and 88/531 (17%) grade 4 astrocytoma (G4A).

**Table 1.** Demographic characteristics of 1367 adult-type diffuse glioma in UCLA cohort stratified by *IDH* mutational status

	*IDH* Wild Type	*IDH* Mutant
All	836	531
Age, years		
Median****	61	37
Range	77	68
Sex		
Male	520 (62%)	317 (60%)
Female	316 (38%)	214 (40%)
Karnofsky Performance Status****		
100^a^	46 (5%)	62 (12%)
90^a^	451 (54%)	384 (72%)
80^a^	222 (27%)	63 (12%)
70^a^	47 (6%)	12 (2%)
≤60^a^	68 (8%)	7 (1%)
Unknown	2 (0%)	3 (1%)
Extent of Resection***		
Biopsy**	154 (18%)	69 (13%)
STR***	305 (36%)	245 (46%)
GTR	370 (44%)	210 (40%)
Unknown	7 (1%)	7 (1%)
Initial diagnosis		
GBM	828 (99%)	–
Gliosarcoma	8 (1%)	–
G2O	–	142 (27%)
G3O	–	68 (13%)
G2A	–	121 (23%)
G3A	–	112 (21%)
G4A	–	88 (17%)
*MGMT* ****		
Methylated	283 (34%)	234 (44%)
Unmethylated	469 (56%)	125 (24%)
Unknown	84 (10%)	172 (32%)
Had RT****	764 (91%)	392 (74%)
Had TMZ****	756 (90%)	328 (62%)

Values in parentheses represent column percentages. Categorical variables were compared with Chi-squared test, and age was compared with Welch’s *t*-test.

** *P*  <  .01.

*** *P*  <  .001.

**** *P*  <  .0001.

a Indicates significant post-hoc Fisher’s exact test results after Bonferroni correction for multiple comparisons.

Abbreviations: STR, sub-total resection; GTR, gross total resection; *MGMT, O6-methylguanine-DNA-methyltransferase*; G2O, grade 2 oligodendroglioma; G3O, grade 3 oligodendroglioma; G2A, grade 2 astrocytoma; G3A, grade 3 astrocytoma; G4A, grade 4 astrocytoma; RT, radiation therapy; TMZ, temozolomide.

We identified 145 *IDH* wild-type and 46 *IDH* mutant patients with MCG (sMCG and mMCG) (Table 2). Additionally, in seven wild-type cases and one mutant case, mMCG arose from sMCG (smMCG). As a result, the sum of the instances of sMCG and mMCG exceed the total number of MCG. Overall, we observed a higher prevalence of MCG among *IDH* wild-type versus mutant patients (WT: 152/836, 18%; Mut: 47/531, 9%, *P*  <  .0001, Fisher’s exact test). When stratified into sMCG and mMCG, we found a higher prevalence of sMCG in the *IDH* wild-type cases than in the mutant cases (WT: 98/836, 13%; Mut: 21/531, 4%, *P*  <  .0001, Fisher’s exact test), and the prevalence of mMCG between *IDH* mutant and wild-type cases was trending (WT: 61/836, 8%; Mut: 27/531, 5%, *P*  =  .06, Fisher’s exact test), although these latter values may depend on the duration of MRI follow-up. The median time of MRI follow-up as defined by the time interval between first and last available MRI is 51.7 months for the *IDH* mutant MCG and 11.2 months for the *IDH* wild-type MCG cohorts. In both *IDH* mutant and wild-type cases, mMCG patients received RT and TMZ more often than non-MCG patients throughout the entire course of treatment (Mut RT: mMCG  =  24/26, 92%, non-MCG  =  350/484, 72%, *P*  =  .02; Mut TMZ: mMCG  =  22/26, 85%, non-MCG  =  292/484, 60%, *P*  =  .003; WT RT: mMCG  =  54/54, 100%, non-MCG  =  619/684, 91%, *P*  =  .03; WT TMZ: mMCG  =  54/54, 100%, non-MCG  =  615/684, 90%, *P*  =  .004, Fisher’s exact test) (Table 2). However, because some patients received TMZ and/or RT only after acquiring mMCG, we separately analyzed rates of initial treatment (data not shown). We found initial rates of RT were largely comparable between non-MCG and mMCG patients for *IDH* mutant cases, as were rates of TMZ between non-MCG and mMCG patients in *IDH* wild-type cases (Mut RT: 21/26, 84%, *P*  =  .3; WT TMZ: 53/54, 98%, *P*  =  .08, Fisher’s exact test). We found rates of TMZ between mMCG and non-MCG in the *IDH* mutant cohort to be different (Mut TMZ: 21/26, 85%, *P*  =  .04), and the rates of RT between mMCG and non-MCG in the *IDH* wild-type cohort were trending (WT RT: 53/54, 98%, *P*  =  .051, Fisher’s exact test). Overall, a higher proportion of *IDH* wild-type glioma patients received RT and TMZ than did *IDH* mutant patients (RT: WT  =  764/836, 91%, Mut= 392/531, 74%, *P*  <  .0001; TMZ: WT= 756/836, 90%, Mut= 328/531, 62%, *P*  <  .0001, Fisher’s exact test) (Table 1).

**Table 2.** Demographic characteristics of 1367 patients of UCLA cohort stratified by multicentricity and *IDH* mutational status

	*IDH* Wild Type				*IDH* Mutant			
	sMCG	mMCG	smMCG	non-MCG	sMCG	mMCG	smMCG	non-MCG
All	91	54	7	684	20	26	1	484
Age								
Median	63	55	60	61	34	37	48	37
Range	61	59	17	77	41	64	–	62
Sex								
Male	53 (58%)	33 (61%)	5 (71%)	429 (63%)	14 (70%)	18 (69%)	1 (100%)	284 (59%)
Female	38 (42%)	21 (39%)	2 (29%)	255 (37%)	6 (30%)	8 (31%)	–	200 (41%)
KPS after initial surgery								
100	4 (4%)	2 (4%)	–	40 (6%)	2 (10%)	3 (12%)	–	57 (12%)
90	43 (47%)	37 (69%)	4 (57%)	367 (54%)	15 (75%)	12 (46%)	–	357 (74%)
80	26 (29%)	14 (26%)	3 (43%)	179 (26%)	2 (10%)	7 (27%)	1 (100%)	53 (11%)
70	7 (8%)	–	–	40 (6%)	–	2 (8%)	–	10 (2%)
≤60	11 (12%)	1 (2%)	–	56 (8%)	1 (5%)	1 (4%)	–	5 (1%)
Unknown	–	–	–	2 (0%)	–	1 (4%)	–	2 (0%)
EOR								
Biopsy	29 (32%)	3 (6%)	1 (14%)	121 (18%)	3 (15%)	5 (19%)	1 (100%)	60 (12%)
STR	31 (34%)	30 (56%)	3 (43%)	241 (35%)	10 (50%)	12 (46%)	–	223 (46%)
GTR	31 (34%)	20 (37%)	3 (43%)	316 (46%)	7 (35%)	8 (31%)	–	195 (40%)
Unknown	–	1 (2%)	–	6 (1%)	–	1 (4%)	–	6 (1%)
Initial diagnosis								
GBM	90 (99%)	53 (98%)	7 (100%)	678 (99%)	–	–	–	–
Gliosarcoma	1 (1%)	1 (2%)	–	6 (1%)	–	–	–	–
G2O	–	–	–	–	3 (15%)	2 (8%)	–	137 (28%)
G3O	–	–	–	–	1 (5%)	4 (15%)	–	63 (13%)
G2A	–	–	–	–	4 (20%)	9 (35%)	1 (100%)	107 (22%)
G3A	–	–	–	–	9 (45%)	5 (19%)	–	98 (20%)
G4A	–	–	–	–	3 (15%)	6 (23%)	–	79 (16%)
*MGMT*								
Methylated	24 (26%)	17 (31%)	3 (43%)	239 (35%)	8 (40%)	14 (54%)	–	212 (44%)
Unmethylated	55 (60%)	33 (61%)	3 (43%)	378 (55%)	4 (20%)	7 (27%)	–	114 (24%)
Unknown	12 (13%)	4 (7%)	1 (14%)	67 (10%)	8 (40%)	5 (19%)	1 (100%)	158 (33%)
Treatment								
Had RT	84 (92%)	54 (100%)	7 (100%)	619 (90%)	18 (90%)	24 (92%)	1 (100%)	350 (72%)
Had TMZ	80 (90%)	54 (100%)	7 (100%)	615 (90%)	13 (65%)	22 (85%)	1 (100%)	292 (60%)

Values in parentheses represent column percentages.

Abbreviations: sMCG, synchronous multicentric glioma; mMCG, metachronous multicentric glioma; smMCG, mMCG arose from sMCG; non-MCG, non-multicentric glioma; STR, subtotal resection; GTR, gross total resection; *MGMT, O6-methylguanine-DNA-methyltransferase*; G2O, grade 2 oligodendroglioma; G3O, grade 3 oligodendroglioma; G2A, grade 2 astrocytoma; G3A, grade 3 astrocytoma; G4A, grade 4 astrocytoma; RT, radiation therapy; TMZ, temozolomide.

### Multicentricity Is Associated With Lower OS in Both IDH Mutant and Wild-Type Patients

**Figure 1.**
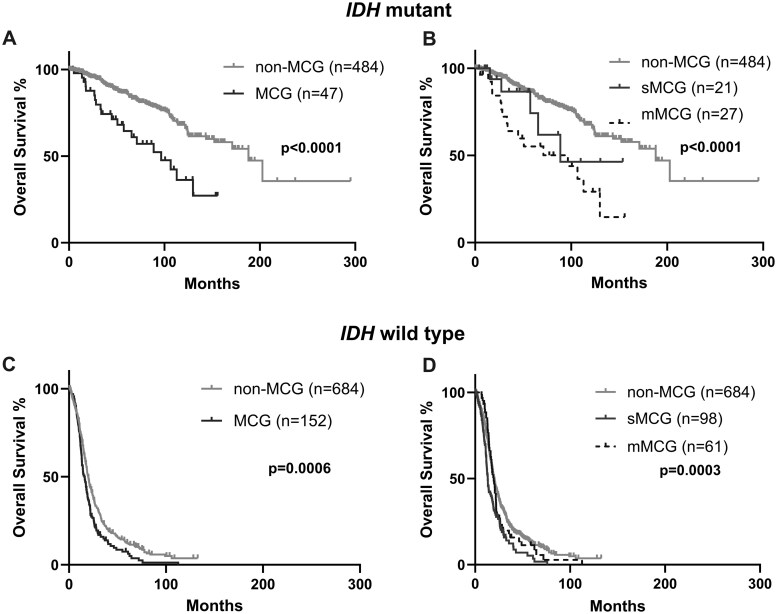
Prognostic impact of MCG on OS. (A-D) Univariate Kaplan-Meier analysis comparing OS between groups of (A) *IDH* mutant MCG versus non-MCG (*P*  <  .0001), (B) *IDH* mutant sMCG, mMCG, and non-MCG (*P*  <  .0001), (C) *IDH* wild-type MCG versus non-MCG (*P*  =  .0006), and (D) *IDH* wild-type sMCG, mMCG, and non-MCG (*P*  =  .0003).

To compare the OS of MCG and non-MCG within *IDH* wild-type and mutant patients, we determined the median OS using KM analysis and confirmed results using Cox-multivariate analysis. In this analysis, we defined smMCG as sMCG and mMCG; these cases were therefore included into both, and the sum of sMCG and mMCG exceeded total MCGs. The median OS of *IDH* mutant MCG cases was 96.6 months, which was shorter than the 188-month median of non-MCG cases (*P*  <  .0001) (Figure 1A, Supplementary Table S4). These findings were confirmed by Cox-multivariate analysis (MCG: HR  =  2.00, *P*  =  .007) (Supplementary Table S2). When we stratified our *IDH* mutant MCG cohort into sMCG and mMCG, OS differed across mMCG, sMCG, and non-MCG (*P*  <  .0001) (Figure 1B). However, post-hoc analysis demonstrated a difference in OS between mMCG, but not sMCG, and non-MCG (mMCG vs non-MCG: *P*  <  .0001; sMCG vs non-MCG: *P*  =  .3) (Supplementary Table S4). These findings were confirmed using multivariate analysis as mMCG but not sMCG was associated with reduced OS when compared to non-MCG (mMCG: HR  =  2.64 *P*  =  .001, sMCG: HR  =  1.22, *P*  =  .7) (Table 3). Additional independent prognostic factors in *IDH* mutant OS include age at diagnosis (HR  =  1.02, *P*  =  .02), which was associated with increased risk of mortality; not receiving radiation therapy (HR  =  0.43, *P*  =  .05) and high KPS after first surgery (HR  =  0.93, *P*  <  .0001) were associated with decreased risk. Only G4A was associated with decreased OS when compared with G2O (G3O: HR  =  0.94, *P*  =  .9; G2A: HR  =  1.34, *P*  =  .4; G3A: HR  =  1.62, *P*  =  .9; G4A: HR  =  3.18, *P*  =  .0008). Overall, mMCG, but not sMCG, was associated with decreased OS for *IDH* mutant glioma patients and appeared to be driven by G4A patient outcomes.

**Table 3.** Cox-multivariate analysis of OS by *IDH* mutational status

	*IDH *Mutant			*IDH *Wild Type		
Variable	HR	*P* value	95% CI	HR	*P* value	95% CI
sMCG	1.22	.7	0.42 to 2.80	1.46	.003	1.13 to 1.86
(ref. non-MCG)						
mMCG	2.64	.001	1.42 to 4.58	1.44	.02	1.05 to 1.93
(ref. non-MCG)						
smMCG	–	–	–	1.06	.9	0.45 to 2.08
(ref. non-MCG)						
Age at Dx	1.02	.02	1.00 to 1.04	1.02	<.0001	1.02 to 1.03
Male (ref. female)	1.33	.2	0.90 to 2.00	1.25	.007	1.06 to 1.47
KPS	0.93	<.0001	0.91 to 0.96	0.97	<.0001	0.96 to 0.98
*MGMT*	1.63	.06	0.99 to 2.69	2.14	<.0001	1.79 to 2.57
Unmethylated						
(ref. methylated)						
Biopsy/STR	1.34	.1	0.91 to 2.02	1.41	<.0001	1.20 to 1.66
(ref. GTR)						
No TMZ	1.01	.98	0.53 to 1.86	1.33	.3	0.79 to 2.17
(ref. received TMZ)						
No RT	0.43	.05	0.18 to 0.95	1.78	.03	1.05 to 2.97
(ref. received RT)						
G3O (ref. G2O)	0.94	.9	0.45 to 1.92	–	–	–
G2A (ref. G2O)	1.34	.4	0.70 to 2.59	–	–	–
G3A (ref. G2O)	1.62	.9	0.83 to 3.24	–	–	–
G4A (ref. G2O)	3.18	.0008	1.63 to 6.36	–	–	–

Abbreviations: sMCG, synchronous multicentric glioma; mMCG, metachronous multicentric glioma; non-MCG, non-multicentric glioma; STR, subtotal resection; GTR, gross total resection; *MGMT, O6-methylguanine-DNA-methyltransferase*; EOR, extent of resection; TMZ, temozolomide; RT, radiation therapy; G2O, grade 2 oligodendroglioma; G3O, grade 3 oligodendroglioma; G2A, grade 2 astrocytoma; G3A, grade 3 astrocytoma; G4A, grade 4 astrocytoma.

For patients within our *IDH* wild-type cohort, the median OS of MCG (15.9 months) was shorter than the median OS of non-MCG (19.9 months) (*P*  =  .0006) (Figure 1C, Supplementary Table S4). This result was confirmed by Cox-multivariate analysis (MCG: HR  =  1.42, *P*  =  .0004) (Supplementary Table S2). As in our *IDH* mutant cohort, we identified differences in OS across sMCG, mMCG, and non-MCG (*P*  =  .0003) (Figure 1D). In our *IDH* wild-type cohort, the median OS for sMCG (13.3 months) was shorter than that of mMCG (19.6 months) (*P*  =  .03) (Supplementary Table S4). Additionally, on post-hoc analysis, sMCG, but not mMCG, had a shorter OS than non-MCG patients (sMCG vs non-MCG: *P*  <  .0001; mMCG vs non-MCG: *P*  =  .4) (Supplementary Table S4). However, on Cox-multivariate analysis, both mMCG and sMCG were found to be associated with lower OS than non-MCG (sMCG: HR  =  1.46, *P*  =  .003, mMCG: HR  =  1.44, *P*  =  .02) (Table 3). Independent prognostic factors associated with increased risk of mortality included older age at diagnosis (HR  =  1.02, *P*  <  .0001), male sex (HR  =  1.25, *P*  =  .007), unmethylated *MGMT* promoter (HR  =  2.14, *P*  <  .0001), subtotal extent of resection (HR  =  1.41, *P*  <  .0001), and not receiving RT (HR  =  1.78, *P*  =  .03). The only factor associated with decreased risk of mortality in the *IDH* wild-type cohort was high KPS after initial surgery (HR  =  0.97, *P*  <  .0001). Overall, based on multivariate analysis, both sMCG and mMCG were associated with shorter OS for *IDH* wild-type glioma patients.

To compare the interval that mMCG cases survived as non-MCG before acquiring MCG, we also calculated residual OS from time of mMCG acquisition. In *IDH* mutant mMCG, the median residual OS starting from time of acquisition was 14.9 months and was shorter than the OS of sMCG (88.7 months) (sMCG: *P*  =  .003) (Supplementary Table S4). In *IDH* wild-type mMCG, the median residual OS was 7.2 months and was similarly shorter compared to both sMCG OS (13.3 months, *P*  <  .0001) (Supplementary Table S4).

To examine interaction effects between MCG types and *IDH* mutational status, we then compared OS between *IDH* mutant and wild type gliomas by analyzing a combined cohort including *IDH* mutant and wild-type cases. As expected, *IDH* wild-type cases had lower OS than mutant cases irrespective of MCG status (non-MCG: *P*  <  .0001, sMCG: *P*  <  .0001, mMCG: *P*  <  .0001) (data not shown). This was confirmed by Cox regression analysis, which found *IDH* mutant status to be an independent prognostic factor (HR  =  0.12, *P*  <  .0001) (Supplementary Table S3). Additionally, we found interaction effects of *IDH* mutational status on OS in mMCG only (sMCG: *IDH* Mut, HR  =  0.93, *P*  =  .9; mMCG: *IDH* Mut, HR  =  2.22, *P*  =  .01; smMCG: *IDH* Mut, HR  =  7.30, *P*  =  .07), confirming that mMCG is associated with decreased OS in *IDH* mutant gliomas.

### IDH Wild-Type Gliomas Acquire Multicentricity More Quickly than IDH Mutant Gliomas

**Figure 2.**
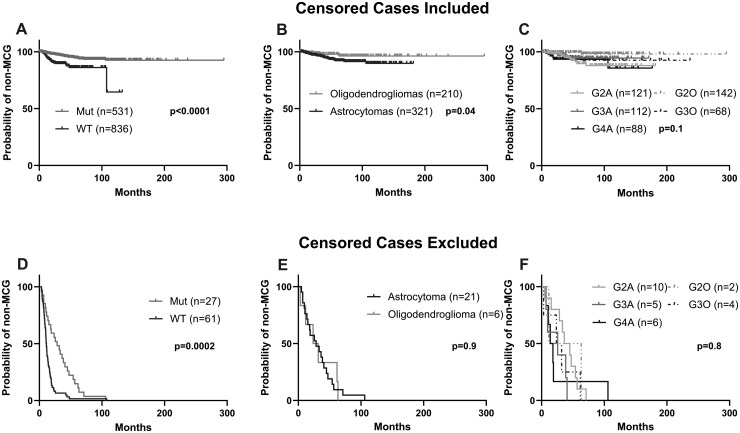
Impact of pathology on TtM. (A-F) Univariate Kaplan-Meier analysis comparing TtM. (A-C) Comparison of TtM in all patients between (A) all *IDH* wild types and mutants (*P*  <  .0001), (B) all astrocytomas and oligodendrogliomas (*P*  =  .04), and (C) all *IDH* mutant diffuse glioma pathologies (*P*  =  .1). (D-F) Comparison of TtM in mMCG patients only (D) in *IDH* mutants versus wild types (*P*  =  .0002), (E) in astrocytomas versus oligodendrogliomas (*P*  =  .9) (F) all *IDH* mutant diffuse glioma pathologies (*P*  =  .8). Initial pathology was used for all analyses. G2O, grade 2 oligodendroglioma; G3O, grade 3 oligodendroglioma; G2A, grade 2 astrocytoma; G3A, grade 3 astrocytoma; G4A, grade 4 astrocytoma.

To compare median time to mMCG (TtM) between *IDH* mutant and wild-type glioma patients, we performed KM analyses on patients stratified by *IDH* mutation (Figure 2A-C) and on those who developed mMCG, excluding censored patients (Figure 2D-F). When censored patients were included, we found TtM to be shorter in the *IDH* wild-type than in the mutant cohorts (*P*  <  .0001) (Figure 2A). This result was confirmed by Cox regression analysis, and we found TtM was longer in the mutant than in the wild-type patients (HR  =  0.38, *P*  =  .005) (Supplementary Table S5). After removing censored patients from the analysis, we again found median TtM to be shorter in *IDH* wild-type patients (11.2 months) than in *IDH* mutant patients (28.4 months) (*P*  =  .0002) (Figure 2D), and Cox regression analysis confirmed this result (HR  =  0.29, *P*  <  .0001) (Supplementary Table S5).

To compare TtM between the different *IDH* mutant pathologies, we performed KM analysis on 321 astrocytoma and 210 oligodendroglioma cases according to initial diagnoses and observed shorter TtM in astrocytomas versus oligodendrogliomas (*P*  =  .04) (Figure 2B). However, Cox-multivariate analysis did not confirm this difference in TtM (HR  =  1.66, *P*  =  .4) (Supplementary Table S6). To look at TtM only among patients who developed mMCG, a comparison of astrocytoma and oligodendroglioma excluding censored patients also revealed no difference in TtM (*P*  =  .9) (Figure 2E), confirmed by Cox-multivariate analysis (HR  =  2.02, *P*  =  .4) (Supplementary Table S6). The sole independent factor impacting TtM in these analyses was high KPS after initial surgery, which was associated with decreased risk of acquiring mMCG (w/censored: HR  =  0.93, *P*  =  .005; mMCG only: HR  =  0.92, *P*  =  .01).

Additional KM analyses were performed on the *IDH* mutant cohort stratified by diagnoses (142 G2O, 68 G3O, 121 G2A, 112 G3A, and 88 G4A) (Figure 2C, Table 1). Additional pairwise analysis demonstrated shorter TtM versus G2O and G2A and G4A only (G2A vs G2O: *P*  =  .007, G3A vs G2O: *P*  =  .1, G4A vs G2O: *P*  =  .01) (Supplementary Table S7). However, while difference in TtM was trending between G2O and G3O (*P*  =  .07), we found no significant differences in TtM between G3O and astrocytoma pathologies (G2A vs G3O: *P*  =  .5, G3A vs G3O: *P*  =  .7, G4A vs G3O: *P*  =  .6), nor between astrocytoma pathologies (G2A vs G3A: *P*  =  .3, G2A vs G4A: *P*  =  .95, G3A vs G4A: *P*  =  .4) (Supplementary Table S7). On Cox regression analysis using G2O as reference, we also did not find any pathology to be an independent risk factor for TtM in the *IDH* mutant cohort (G3O: HR  =  1.48, *P*  =  .7; G2A: HR  =  2.93, *P*  =  .2; G3A: HR  =  1.44, *P*  =  .7; G4A: HR  =  1.98, *P*  =  .4) (Table 4). The only factor that decreased risk of acquiring mMCG in *IDH* mutants was high KPS after initial surgery (HR  =  0.93, *P*  =  .006). Furthermore, analysis of only mMCG separated by diagnosis revealed no difference in TtM between these groups (*P*  =  .8) (Figure 2F). This is confirmed by multivariate analysis using G2O as reference (G3O: HR  =  0.09, *P*  =  .3; G2A: HR  =  0.14, *P*  =  .4; G3A: HR  =  2.31, *P*  =  .8; G4A: HR= 0.71, *P*  =  .7) (Table 4). Overall, these results indicate that *IDH* wild-type patients acquired MCG faster than mutants when including or excluding censored patients, and within the *IDH* mutant groups, pathological subtypes did not influence TtM.

**Table 4.** Cox multivariate analysis of TtM in *IDH* mutant glioma patients stratified by pathology with and without patients censored in their TtM

	mMCG with Censored			mMCG Only		
Variable	HR	*P* value	95% CI	HR	*P* value	95% CI
Age at Dx	1.02	.2	0.99 to 1.06	1.01	.8	0.94 to 1.09
Male	1.71	.2	0.72 to 4.54	1.19	.8	0.26 to 4.73
(ref. female)						
KPS	0.93	.006	0.89 to 0.98	0.97	.4	0.89 to 1.04
*MGMT *unmethylated	0.86	.8	0.29 to 2.37	0.05	.002	0.01 to 0.31
(ref. methylated)						
GTR	0.79	.6	0.32 to 1.80	1.61	.5	0.37 to 6.88
(ref. Biopsy/STR)						
No TMZ	0.39	.2	0.06 to 1.58	0.01	.2	2.02e−005 to 6.44
(ref. received TMZ)						
No RT	0.18	.2	0.01 to 1.44	2.77e + 016	>.99	–
(ref. received RT)						
Initial G3O	1.48	.7	0.27 to 11.19	0.09	.3	0.0007 to 9.51
(ref. G2O)						
Initial G2A	2.93	.2	0.65 to 20.64	0.14	.4	0.0007 to 21.11
(ref. G2O)						
Initial G3A	1.44	.7	0.28 to 10.79	2.31	.8	–
(ref. G2O)						
Initial G4A	1.98	.4	0.40 to 14.76	0.71	.7	0.004 to 65.81
(ref. G2O)						

Abbreviations: MGMT, *O6-methylguanine-DNA-methyltransferase*; G2O, grade 2 oligodendroglioma; G3O, grade 3 oligodendroglioma; G2A, grade 2 astrocytoma; G3A, grade 3 astrocytoma; G4A, grade 4 astrocytoma.

### Anatomical Distribution of MCG Tumors in an Individual Patient Indicates That Tumors can be Supratentorial/Unilateral, Supratentorial/Infratentorial, Supratentorial/Contralateral, and Infratentorial/Infratentorial

To characterize the spatial distributions of tumors within an individual patient, we separated MCG patients into paired MCG with two lesions only (138/199) (Supplementary Table S8) and MCG with ≥3 lesions (61/199) (Supplementary Table S9). The proportion of paired MCG is 37/47 (79%) among *IDH* mutant MCG and 101/152 (66%) among *IDH* wild-type MCG, and for ≥3 lesions is 10/47 (21%) among *IDH* mutant MCG and 51/152 (34%) among *IDH* wild-type MCG. The ratio of paired versus ≥3 lesions was similar between *IDH* mutant and wild-type cohorts (*P*  =  .1, Fisher’s exact test).

We first tabulated the distribution of lesions per lobe and region of the brain in addition to the combination of regions in each patient; however, to increase statistical power, we collapsed these separate combinations into larger groups for analysis (Supplementary Table S8). We analyzed loci combinations in paired sMCG and mMCG as supratentorial/infratentorial (one lesion in the cerebrum and one lesion in the brainstem, cerebellum, and/or spinal cord), infratentorial/infratentorial (both lesions infratentorial), supratentorial/bilateral (one lesion in each hemisphere of the cerebrum), or supratentorial/unilateral (both lesions in the unilateral cerebrum). In paired *IDH* mutant sMCG, we observed 11/17 (65%) as supratentorial with unilateral involvement only, and none demonstrated infratentorial involvement; however, *IDH* wild-type sMCG demonstrated distribution between these four subgroups (10/64, 16%; 1/64, 2%; 29/64, 45%; and 24/64, 38%, respectively) (Supplementary Table S8). Although we did not find the distribution of tumor loci between paired *IDH* mutant and wild type to be different (*P*  =  .2, Fisher’s exact test), both *IDH* mutant and wild-type cohorts demonstrated higher proportions of infratentorial tumor loci in mMCG than in sMCG (Mut: 13/20, 67% vs 0/17, 0%, *P*  <  .0001; WT: 18/37, 49% vs 11/64, 17%, *P*  =  .001, Fisher’s exact test). Interestingly, among *IDH* mutant and wild-type mMCG with supratentorial/infratentorial pairings, all initial tumors were supratentorial, with infratentorial loci appearing later over time.

In MCG with ≥3 tumors, we similarly tabulated loci combinations as supratentorial/infratentorial (at least one lesion in the cerebrum and one lesion in the brainstem, cerebellum, and/or spinal cord), supratentorial/contralateral (at least one lesion in each hemisphere of the cerebrum), supratentorial/unilateral (all lesions in the unilateral cerebrum) or infratentorial/infratentorial (all lesions infratentorial) (Supplementary Table S9). As smMCG involves development of additional tumor(s) to sMCG, all cases necessarily included ≥3 lesions. Interestingly, *IDH* mutant MCG with ≥3 lesions had a higher prevalence of infratentorial involvement than did the *IDH* wild-type cohort (Mut: 8/10, 80%, WT: 17/51, 33%, *P*  =  .01, Fisher’s exact test). Additionally, in *IDH* wild-type MCG with ≥3 lesions, mMCG also have more infratentorial distribution than do sMCG (mMCG  =  15/24, 63%, sMCG  =  5/34, 15%, *P*  =  .0002, Fisher’s exact test). Notably, in *IDH* wild-type MCG, there were three patients with intracranial glioblastoma with additional pathologically confirmed tumor foci outside the CNS (Supplementary Table S10).

### Resected Lesions Show Discordant Pathologies Within Our Cohort

To describe the concordance of tumors with confirmed pathologies within individual MCG patients, we identified all MCG patients with more than one resected locus. We found that MCG uncommonly have multiple resected lesions. Of 152 total *IDH* wild-type MCG, 28 (18%) had resection of more than one lesion, and of 47 *IDH* mutant MCG, 7 (15%) had double-resection (Supplementary Table S11). In either cohort, we did not identify any patient with >2 lesions resected throughout the course of treatment. In *IDH* mutant but not wild-type patients, we observed a difference between the proportions of sMCG and mMCG with double-resection (Mut: sMCG  =  6/20, 30%, mMCG= 1/26, 4%, *P*  =  .03; WT: sMCG  =  16/91, 18%, mMCG  =  12/54, 22%, *P*  =  .8, Fisher’s exact test).

Notably, in double-resected *IDH* wild-type sMCG and mMCG cases, pathology was uniformly concordant between lesions (sMCG: 16/16, mMCG 12/12). In contrast, among *IDH* mutant cases, the one double-resected mMCG case showed concordant pathology, whereas 6/7 sMCG were discordant, exhibiting different pathologies between lesions in the same patient (Supplementary Table S11). Of the double-resected sMCG, 2 had combinations of oligodendroglioma and astrocytoma (one G3A/G3O and one G3A/G2O), and one was a combination of pilocytic astrocytoma and G3O; the other two had combinations of G2A and G3A; and interestingly, the sole concordant case had different *IDH* mutations in each lesion (R132H and R132S). A few initial MRIs of sMCG cases with discordant pathologies are shown in Supplementary Figure S2.

## Discussion

Multicentric glioma (MCG) is a radiographically defined subset of gliomas that can occur synchronously (sMCG) and/or metachronously (mMCG). In this retrospective study of 836 *IDH* wild-type and 531 *IDH* mutant glioma patients, we expand on current understanding of MCG. MCG has higher prevalence in *IDH* wild-type gliomas than in *IDH* mutant gliomas. While both subgroups of MCG are associated with lower OS in *IDH* wild-type glioma patients, only mMCG exhibited prognostic value in *IDH* mutant patients. TtM is longer in the *IDH* mutant cohort than in the *IDH* wild-type cohort in analyses that included all patients and in analyses restricted only to patients who developed mMCG. In both *IDH* mutant and wild-type MCG, mMCG was associated with greater infratentorial involvement than sMCG, and when comparing MCG with ≥3 lesions, we found the prevalence of infratentorial involvement to be higher in the *IDH* mutant than in the wild-type cohort. Among double-biopsied MCG, we found discordance in pathology between lesions of the same patient in 5/7 of *IDH* mutant and 0/28 of *IDH* wildtype glioma patients, suggesting that multiple lesions should be biopsied in cases of *IDH* mutant MCG. These results demonstrate differences in MCG characteristics between *IDH* wild-type and mutant cohorts.

In terms of prevalence, MCG (sMCG, mMCG, and smMCG) in *IDH* wild type occurred at 18% and in *IDH* mutant at 9%. We observed this pattern in sMCG and mMCG groups independently, and both subgroups of MCG had lower prevalence in the *IDH* mutant than in the wild-type cohorts. Our results are within previously documented ranges in the literature (1%-35%^3^^,^^9^ and 2%-10%^6^ for *IDH* wildtype and mutant MCG, respectively). We suspect that the mMCG prevalence is underestimated preferentially in the *IDH* mutant cohort due to incomplete imaging follow-up. Nonetheless, both median follow-up intervals exceed median TtM (excluding censored patients) (Supplementary Figure S3). In support of previous studies,^3^^,^^11^ OS was lower in MCG than in non-MCG in the *IDH* wild-type glioblastoma. As expected, we found age at diagnosis, *MGMT* methylation status, extent of resection, KPS, and *IDH* mutational status were all independent prognostic factors for OS within our cohort.^2^^,^^6^^,^^12^^,^^13^^,^^16^^,^^20^ Additionally, we found sex to be a prognostic factor in the *IDH* wild-type cohort only, with male sex associated with poorer OS. We also compared TtM on univariate analysis in *IDH* wild type and mutant and found that, as expected, wild type had shorter TtM than *IDH* mutant on average.

Interestingly, our study found that in the *IDH* mutant cohort, sMCG and mMCG had different prognoses. Our findings suggest that initial presentation with MCG (sMCG) is not a negative prognostic factor while acquired MCG (mMCG) was associated with worse prognosis. This discrepancy contradicts findings from Tariq et al. who found that low grade gliomas with mMCG had longer OS than solitary glioma and had comparable survival to sMCG once ectopic recurrence occurs.^6^ Additionally, interaction analysis of MCG status and *IDH* mutational status on OS found interaction effects between *IDH* mutation and mMCG only, further confirming that only mMCG is associated with decreased OS in the *IDH* mutant cohort. Overall, our results suggest that in *IDH* mutant cases, mMCG is a clinical marker of accelerated disease, and more aggressive treatment of these cases should be considered when clinically feasible.

We tabulated the distributions of sMCG and mMCG foci separately. We observed that the majority of sMCG for *IDH* mutant gliomas were supratentorial/unilateral. For mMCG, we found high rates of supratentorial/infratentorial pairs, especially in *IDH* mutant cases. While *IDH* wild-type mMCG with infratentorial spread from a primary supratentorial tumor location has been described before, it is a rare and severe recurrence type, occurring only in 6/134 (5%) of patients.^21^ Similarly, *IDH* mutant MCG with infratentorial/supratentorial pairing has only been described in two case reports.^22^^,^^23^ However, the results from our cohort suggest that MCG with infratentorial spread from a primary supratentorial tumor may be more common than previously reported, especially in *IDH* mutant mMCG. Therefore, given the number of MCG with infratentorial lesions, obtaining imaging of the spinal cord should be considered to determine true tumor burden.

Additionally, we examined the pathological concordance between tumor foci in MCG cases where two or more tumor foci were biopsied and found 100% concordance in diagnosis between foci in *IDH* wild-type patients. However, we found that in 5/7 *IDH* mutant cases where two or more foci were biopsied, the patient had discordant pathologies. In the literature, there has been one previously described case of an oligodendroglioma patient with incongruous *IDH1* mutations between lesions.^23^ We found a similar case of incongruous *IDH* mutations between MCG tumor loci in an astrocytoma patient. As *IDH* mutations are considered early events in gliomagenesis,^24^ the incongruities found between lesions points to independent origin of these MCG tumors. These results indicate that more extensive surgical strategies should be considered when clinically appropriate in *IDH* mutant MCG patients, as discordance between tumor pathologies may significantly alter management. For example, one sMCG patient in our cohort was found to have a left frontal lobe G3O tumor and a right frontal lobe pilocytic astrocytoma tumor. Due to this discovery, the treatment team was able to defer radiation to the right frontal lesion. Had the secondary pilocytic astrocytoma not been resected and pathology between lesions been assumed to be concordant, the field of radiation would have been unnecessarily expanded, leading to increased risk of morbidities associated with treatment. Additionally, with the recent approval of vorasidenib for treatment-naïve *IDH* mutant grade 2 gliomas, patients initially diagnosed with G2O or G2A but with a remote multicentric tumor of a higher grade may warrant additional treatment. As such, in many instances of discordant pathologies in our cohort, the identity of the second lesion warranted additional clinical consideration.

Despite our large cohort, the results of this study will need to be validated by other retrospective cohorts. Examination of the true prevalence of mMCG is limited by the follow-up imaging time, and patients who were considered non-MCG or sMCG only could develop mMCG past the censor date. Especially in *IDH* mutant patients, short MRI follow-up time (median 51.7 months) compared to OS (median 188.16 months) could explain the lower prevalence of MCG compared to *IDH* wild-type cases. In the case of mMCG, although new tumor foci are often remote from the initial site, it is unknown whether the new and the primary foci hyperintensities would have overlapped had the primary tumor not been resected. Moreover, within the *IDH* mutant cohort, we found no difference in OS between pathologies on multivariate analysis besides G2O versus G4A (Table 3). However, while not statistically significant, the hazard ratios aligned with expected trends, with G3O showing the lowest and G4A the highest with respect to G2O. As our study relies on available patient data only, not all patients in the cohort had *MGMT* promoter methylation status*, CDKN2A/B* loss, or *1p/19q* codeletion testing, which led to uneven application of diagnostic criteria. Therefore, not all patients were included based on WHO 2021 guidelines. Additionally, not all sMCG cases with discordant pathologies had full molecular and NGS characterization, limiting our ability to analyze differences in greater depth. For MCG without a second biopsy, it is possible that the secondary lesion is nonspecific. However, our imaging criteria for inclusion (i.e., steady growth over time, persistence through treatment) are characteristic of tumor rather than nonspecific lesions, such as ischemic disease or treatment effect. Furthermore, in mMCG patients, the high prevalence of infratentorial distribution may be related to ventricular breach during surgery, and future studies could investigate the role of this factor in the development of infratentorial mMCG. Finally, selection bias may have contributed to the discordant pathologies observed in *IDH* mutant MCG, as re-resection may have been attempted only when the second lesion appeared atypical.

In conclusion, this study identified MCG as a subgroup of glioma that was more common in *IDH* wild-type patients than in mutant patients. MCG has lower OS than non-MCG in both *IDH* mutant and wild-type patients. When stratified into sMCG and mMCG, we found that, while both sMCG and mMCG were associated with decreased OS in the *IDH* wild-type cohort, only mMCG is associated with decreased OS in the *IDH* mutant cohort. We found that TtM was the shortest in glioblastoma/gliosarcoma and longest in G2O. A high proportion of mMCGs were classified as infratentorial/supratentorial pairings, especially in *IDH* mutant MCG. Finally, we found high levels of pathology discordance between resected tumor loci in *IDH* mutant cases. This leads to the recommendation to attempt tissue confirmation in *IDH* mutant MCG cases when feasible. Further research could examine the role of distance between MCG lesions, compare MCG and multifocal glioma, and apply more sophisticated descriptive analyses to imaging distribution of MCG. Additionally, studies quantifying the molecular markers of MCG, especially the molecular differences between discordant *IDH* mutant sMCG, could help determine a potential genotype more predisposed to developing MCG. Furthermore, assessing the relationship between radiation dosimetry, extent of resection, ventricle breach, and acquiring mMCG could be helpful in predicting and preventing mMCG, which has unfavorable prognostic outcomes in both *IDH* mutant and wild-type cases.

## Supplementary Material

vdag047_Supplementary_Data

## Data Availability

Data will be made available upon reasonable request to the corresponding author.
