# Supplementary material for: A single-institution retrospective study of multicentric gliomas stratified by IDH mutational status
Source: Neurooncol Adv. 2026 May 10;8(1):vdag047. doi: 10.1093/noajnl/vdag047 (PMC13157338; doi:10.1093/noajnl/vdag047)
Supplement: vdag047_Supplementary_Data [file vdag047_supplementary_data.docx]

**Supplementary Figure 1:** Simplified algorithm for determining sMCG and mMCG from available patient data and imaging studies.


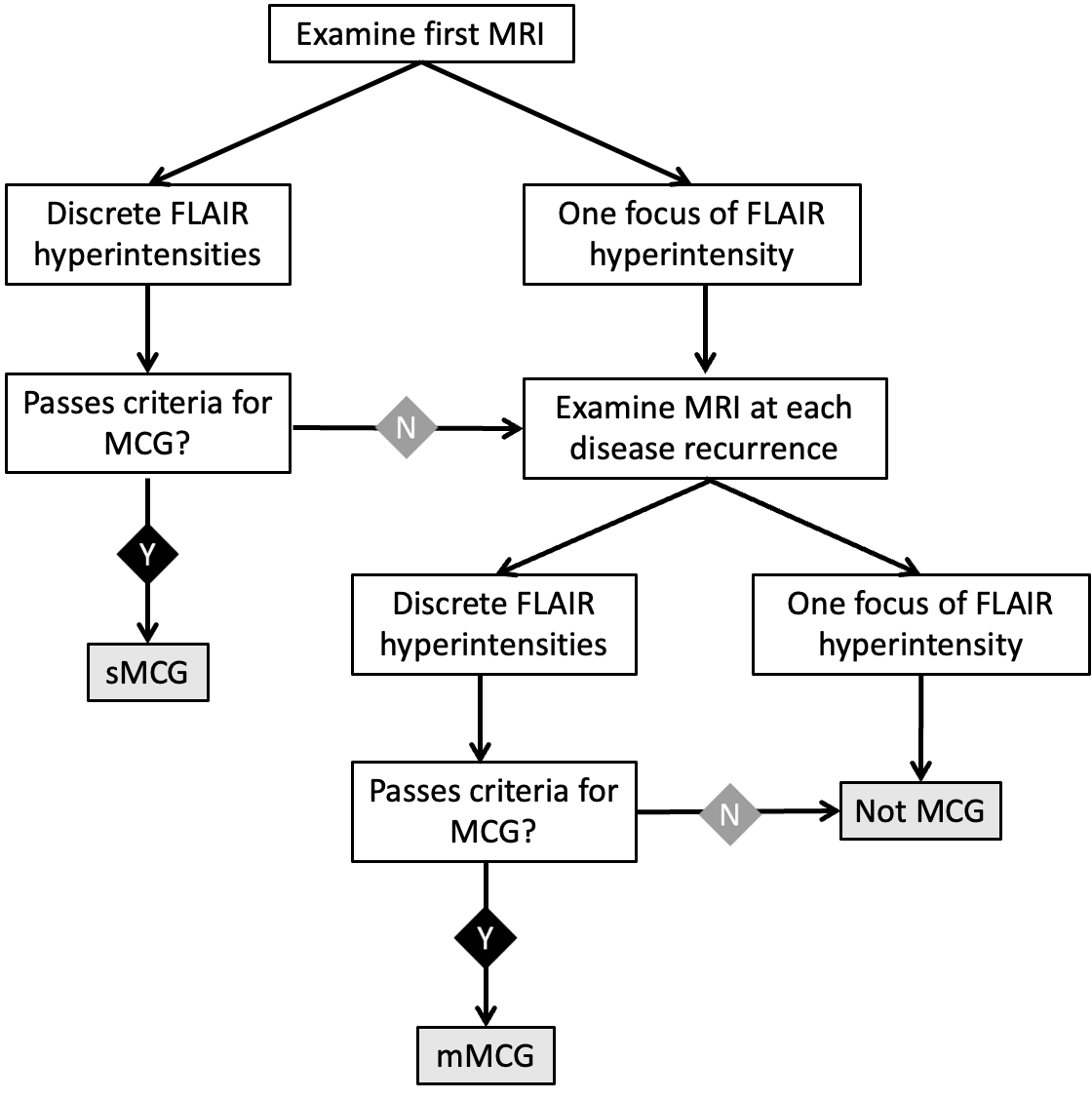


**Supplementary Table 1:**Determination of pathology in *IDH*mutant cohort. G2A, grade 2 astrocytoma; G3A, grade 3 astrocytoma; G4A, grade 4 astrocytoma; G2O, grade 2 oligodendroglioma; G3O, grade 3 oligodendroglioma.

| **Initial Diagnosis** | **WHO 2021**  **(Group 1)** | **WHO 2016**  **(Group 2)** | **Inferred WHO 2021/2016**  **(Group 3)** | **Histological Only**  **(Group 4)** | **Total** |
| --- | --- | --- | --- | --- | --- |
| **G2O** | 139 | - | 2/0 | 1 | 142 |
| **G3O** | 64 | - | 2/0 | 2 | 68 |
| **G2A** | 56 | 46 | 10/3 | 6 | 121 |
| **G3A** | 59 | 32 | 12/3 | 6 | 112 |
| **G4A** | 43 | 11 | 23/8 | 3 | 88 |

**Supplementary Table 2:**Cox-multivariate analysis of OS by *IDH* mutational status. MCG, multicentric glioma; non-MCG, non-multicentric glioma; STR, subtotal resection; GTR, gross total resection; *MGMT, O6-methylguanine-DNA-methyltransferase*; EOR, extent of resection; TMZ, temozolomide; RT, radiation therapy; G2O, grade 2 oligodendroglioma; G3O, grade 3 oligodendroglioma; G2A, grade 2 astrocytoma; G3A, grade 3 astrocytoma; G4A, grade 4 astrocytoma.

|  | ***IDH*Mutant** | | | ***IDH*Wild Type** | | |
| --- | --- | --- | --- | --- | --- | --- |
| **Variable** | **HR** | **P-value** | **95% CI** | **HR** | **P-value** | **95% CI** |
| **MCG**  (ref. non-MCG) | 2.00 | 0.007 | 1.17 to 3.25 | 1.42 | 0.0004 | 1.17 to 1.73 |
| **Age at Dx** | 1.02 | 0.02 | 1.00 to 1.04 | 1.02 | <0.0001 | 1.02 to 1.03 |
| **Male**  (ref. female) | 1.39 | 0.1 | 0.94 to 2.09 | 1.25 | 0.008 | 1.06 to 1.47 |
| **KPS** | 0.93 | <0.0001 | 0.91 to 0.96 | 0.97 | <0.0001 | 0.96 to 0.98 |
| ***MGMT***  **Unmethylated**  (ref. methylated) | 1.71 | 0.04 | 1.04 to 2.81 | 2.16 | <0.0001 | 1.81 to 2.59 |
| **GTR**  (ref. Biopsy/STR) | 0.76 | 0.2 | 0.51 to 1.13 | 0.71 | <0.0001 | 0.60 to 0.84 |
| **No TMZ**  (ref. received TMZ) | 0.91 | 0.8 | 0.47 to 1.67 | 1.33 | 0.3 | 0.79 to 2.17 |
| **No RT**  (ref. received RT) | 0.45 | 0.06 | 0.18 to 0.995 | 1.77 | 0.03 | 1.05 to 2.97 |
| **G3O**(ref. G2O) | 0.99 | 0.97 | 0.48 to 2.01 | - | - | - |
| **G2A**(ref. G2O) | 1.27 | 0.5 | 0.66 to 2.47 | - | - | - |
| **G3A**(ref. G2O) | 1.52 | 0.2 | 0.78 to 3.03 | - | - | - |
| **G4A**(ref. G2O) | 2.84 | 0.002 | 1.47 to 5.65 | - | - | - |

**Supplementary Table 3:**Cox regression analysis of OS combining *IDH*mutant and wild type cases and interaction effects between *IDH*mutational status and MCG status. sMCG, synchronous multicentric glioma; mMCG, metachronous multicentric glioma; smMCG, metachronous arising from synchronous multicentric glioma; STR, subtotal resection; GTR, gross total resection; *MGMT, O6-methylguanine-DNA-methyltransferase*; EOR, extent of resection; TMZ, temozolomide; RT, radiation therapy.

| **Variable** | **HR** | **P-value** | **95% CI** |
| --- | --- | --- | --- |
| **sMCG**(ref. non-MCG) | 1.45 | 0.004 | 1.12 to 1.84 |
| **mMCG**(ref. non-MCG) | 1.41 | 0.02 | 1.04 to 1.87 |
| **smMCG**(ref. non-MCG) | 1.05 | 0.9 | 0.45 to 2.05 |
| **Age at Dx** | 1.02 | <0.0001 | 1.02 to 1.03 |
| **Male**(ref. female) | 1.26 | 0.003 | 1.08 to 1.46 |
| **KPS** | 0.96 | <0.0001 | 0.96 to 0.97 |
| ***MGMT* Unmethylated**  (ref. methylated) | 2.18 | <0.0001 | 1.85 to 2.59 |
| **GTR (**ref. Biopsy/STR) | 0.73 | <0.0001 | 0.62 to 0.84 |
| **No TMZ**(ref. receivedTMZ) | 0.97 | 0.9 | 0.66 to 1.38 |
| **No RT**(ref. received RT) | 1.44 | 0.07 | 0.97 to 2.13 |
| ***IDH*mutant**(ref. *IDH*wild type) | 0.12 | <0.0001 | 0.09 to 0.15 |
| **Interaction Effects** |  |  |  |
| sMCG:*IDH*Mutant | 0.93 | 0.9 | 0.28 to 2.33 |
| mMCG:*IDH*Mutant | 2.22 | 0.01 | 1.13 to 4.09 |
| smMCG:*IDH*Mutant | 7.30 | 0.07 | 0.39 to 42.32 |

**Supplementary Table 4:**Median overall survival (OS) of Kaplan-Meier curves in figure 1 and median residual OS of mMCG with pair-wise analysis of each MCG subtype.

|  | ***IDH*mutant** | | ***IDH*wild type** | |
| --- | --- | --- | --- | --- |
|  | **Median OS (Months)** | **P-value** | **Median OS** **(Months)** | **P-value** |
| **MCG** [vs non-MCG] | 96.6 | <0.0001 | 15.9 | 0.0006 |
| **sMCG**[vs mMCG] | 88.7 | 0.2 | 13.3 | 0.03 |
| **mMCG**[vs non-MCG] | 96.6 | <0.0001 | 19.6 | 0.4 |
| **non-MCG** [vs sMCG] | 188 | 0.3 | 19.9 | <0.0001 |
| **Residual mMCG**[vs sMCG] | 14.9 | 0.003 | 7.2 | <0.0001 |

**Supplementary Table 5:** Cox-multivariate analysis of TtM stratified by *IDH*mutational status. STR, subtotal resection; GTR, gross total resection; *MGMT, O6-methylguanine-DNA-methyltransferase*; EOR, extent of resection; TMZ, temozolomide; RT, radiation therapy.

|  | **mMCG with Censored** | | | **mMCG Only** | | |
| --- | --- | --- | --- | --- | --- | --- |
| **Variable** | **HR** | **P-value** | **95% CI** | **HR** | **P-value** | **95% CI** |
| **Age at Dx** | 0.99 | 0.2 | 0.97 to 1.01 | 0.995 | 0.6 | 0.98 to 1.01 |
| **Male**  (ref. female) | 1.20 | 0.5 | 0.75 to 1.87 | 0.75 | 0.2 | 0.47 to 1.22 |
| **KPS** | 1.00 | 0.8 | 0.97 to 1.03 | 0.94 | 0.0008 | 0.91 to 0.98 |
| ***MGMT***  **Unmethylated**  (ref. methylated) | 1.34 | 0.2 | 0.84 to 2.17 | 1.01 | 0.95 | 0.64 to 1.64 |
| **GTR**  (ref. Biopsy/STR) | 0.65 | 0.08 | 0.40 to 1.05 | 1.04 | 0.89 | 0.64 to 1.66 |
| **No TMZ**  (ref. Received TMZ) | 0.29 | 0.1 | 0.04 to 1.17 | 1.11 | 0.9 | 1.16 to 4.97 |
| **No RT**  (ref. received RT) | 0.41 | 0.5 | 0.02 to 2.99 | 26.9 | 0.008 | 1.68 to 812.00 |
| ***IDH*Mutant**  (ref. wild type) | 0.38 | 0.005 | 0.19 to 0.74 | 0.29 | <0.0001 | 0.15 to 0.54 |

**Supplementary Table 6:** Cox-multivariate analysis of TtM of *IDH*mutant cases only, stratified by histology. STR, subtotal resection; GTR, gross total resection; *MGMT, O6-methylguanine-DNA-methyltransferase*; EOR, extent of resection; TMZ, temozolomide; RT, radiation therapy.

|  | **mMCG with Censored** | | | **mMCG Only** | | |
| --- | --- | --- | --- | --- | --- | --- |
| **Variable** | **HR** | **P-value** | **95% CI** | **HR** | **P-value** | **95% CI** |
| **Age at Dx** | 1.03 | 0.2 | 0.99 to 1.06 | 1.03 | 0.2 | 0.98 to 1.08 |
| **Male**  (ref. female) | 1.89 | 0.2 | 0.80 to 4.95 | 0.42 | 0.2 | 0.12 to 1.42 |
| **KPS** | 0.93 | 0.005 | 0.89 to 0.98 | 0.92 | 0.01 | 0.86 to 0.98 |
| ***MGMT***  **Unmethylated**  (ref. methylated) | 0.87 | 0.8 | 0.30 to 2.32 | 0.20 | 0.02 | 0.05 to 0.70 |
| **GTR**  (ref. Biopsy/STR) | 0.73 | 0.5 | 0.30 to 1.67 | 0.99 | 0.99 | 0.33 to 2.87 |
| **No TMZ**  (ref. Received TMZ) | 0.42 | 0.3 | 0.06 to 1.69 | 0.52 | 0.6 | 0.02 to 6.91 |
| **No RT**  (ref. received RT) | 0.19 | 0.2 | 0.01 to 1.47 | 5.26e+013 | >0.99 | - |
| **Astro**  (ref. Oligo) | 1.66 | 0.4 | 0.57 to 5.42 | 2.02 | 0.4 | 0.47 to 11.84 |

**Supplementary Table 7:**Results of post-hoc analysis of Kaplan-Meier curves shown in Figures 2c (left) and 2f (right). All significance values are given by Log-rank (Mantel-Cox) test. G2O, grade 2 oligodendroglioma; G3O, grade 3 oligodendroglioma; G2A, grade 2 astrocytoma; G3A, grade 3 astrocytoma; G4A, grade 4 astrocytoma.

|  | **Censored Included** | **mMCG Only** |
| --- | --- | --- |
| **G2O with:** | | |
| **G3O** | 0.07 | 0.4 |
| **G2A** | 0.007 | 0.8 |
| **G3A** | 0.1 | 0.3 |
| **G4A** | 0.01 | 0.9 |
| **G2A with:** | | |
| **G3O** | 0.5 | 0.6 |
| **G3A** | 0.3 | 0.09 |
| **G4A** | 0.95 | 0.5 |
| **G3A with:** | | |
| **G3O** | 0.7 | 0.7 |
| **G4A** | 0.4 | 0.9 |
| **G3O with:** | | |
| **G4A** | 0.6 | 0.7 |

**Supplemental Table 8:** Imaging characteristics of 138 MCG patients with paired lesions only in UCLA cohort stratified by *IDH* mutational status and MCG subtype. CC, corpus callosum; F, frontal lobe; P, parietal lobe; O, occipital lobe; T, temporal lobe.

|  | ***IDH*Mutant** | | ***IDH*Wild Type** | |
| --- | --- | --- | --- | --- |
|  | **sMCG** | **mMCG** | **sMCG** | **mMCG** |
| **Total** | 17 | 20 | 64 | 37 |
| **Supra/Infratentorial** | - | 13 (65%) | 10 (16%) | 17 (46%) |
| **Frontal** | - | 7 | 2 | 3 |
| **Parietal** | - | 3 | 5 | 3 |
| **Temporal** | - | 2 | 3 | 5 |
| **Occipital** | - | 1 | - | - |
| **Other (Including thalamus and CC)** | - | - | - | 6 |
| **Infra/Infratentorial** | - | - | 1 (2%) | 1 (3%) |
| **Supratentorial/Bilateral** | 6 (35%) | 5 (25%) | 29 (45%) | 4 (11%) |
| **F/F** | 3 | - | 12 | - |
| **F/P** | - | 2 | 2 | 1 |
| **F/T** | 2 | - | 1 | 1 |
| **F/O** | - | - | 1 | - |
| **F/other** | - | 1 | 5 | - |
| **P/P** | - | - | - | - |
| **P/T** | - | - | 2 | 2 |
| **P/O** | - | - | - | - |
| **P/other** | 1 | - | 3 | - |
| **T/T** | - | 1 | 2 | - |
| **T/O** | - | - | - | - |
| **T/other** | - | 1 | 1 | - |
| **Other patterns** | - | - | - | - |
| **Supratentorial/Unilateral** | 11 (65%) | 2 (10%) | 24 (38%) | 15 (41%) |
| **F/F** | 1 | - | 5 | - |
| **F/P** | 3 | - | 6 | 3 |
| **F/T** | 1 | 2 | 1 | 7 |
| **F/O** | - | - | 2 | 2 |
| **F/other** | 3 | - | 1 | 1 |
| **P/P** | - | - | - | - |
| **P/T** | 2 | - | 4 | - |
| **P/O** | - | - | 1 | 1 |
| **P/other** | - | - | 2 | - |
| **T/T** | - | - | - | - |
| **T/O** | - | - | - | - |
| **T/other** | 1 | - | 2 | 1 |
| **Other patterns** | - | - | - | - |

**Supplemental Table 9:** Imaging characteristics of 61 MCG patients with ≥3 lesions only in UCLA cohort stratified by *IDH*mutational status and MCG subtype.

|  | ***IDH*Mutant** | | | ***IDH*Wild Type** | | |
| --- | --- | --- | --- | --- | --- | --- |
|  | **sMCG** | **mMCG** | **smMCG** | **sMCG** | **mMCG** | **smMCG** |
| **Total** | 3 | 6 | 1 | 27 | 17 | 7 |
| **Supratentorial/**  **Infratentorial** | 3 (100%) | 4 (67%) | 1 (100%) | 2 (7%) | 11 (65%) | 3 (43%) |
| **Infratentorial/**  **Infratentorial** | - | - | - | - | 1 (6%) | - |
| **Supratentorial/**  **Bilateral** | - | 2 (33%) | - | 18 (67%) | 5 (29%) | 4 (57%) |
| **Supratentorial/**  **Unilateral** | - | - | - | 7 (26%) | - | - |

**Supplementary Table 10:**Tabulated locations of MCG patients with extracranial metastasis of *IDH*wild-type GBM confirmed by biopsy.

| **Intracranial Location** | **Extracranial Location** | **Confirmed by Biopsy** |
| --- | --- | --- |
| Left Parietal, Right Temporal | L3 bone  Left Pelvis | Yes |
| Right Parietal | Liver | Yes |
| Left Tentorium Cerebelli | Left Humerus, Left shoulder  Right Posterior Iliac, Left head of femur,  T6 Vertebra | Yes  No |

**Supplementary Table 11:** Pathology concordance of MCG with two or more resected loci stratified by *IDH* mutational status and synchronicity. *^a^* Patient has *IDH1* mutation mismatch between tumors (R132H and R132S)

|  | ***IDH*Mutant** | | ***IDH*Wild Type** | |
| --- | --- | --- | --- | --- |
|  | **sMCG** | **mMCG** | **sMCG** | **mMCG** |
| **>1 Resected Loci** | 6 (33%) | 1 (4%) | 16 (18%) | 12 (22%) |
| **Same Pathology** | 1*^a^* | 1 | 16 | 12 |
| **Same histology, different grade** | 2 | - | - | - |
| **Different histology** | 3 | - | - | - |


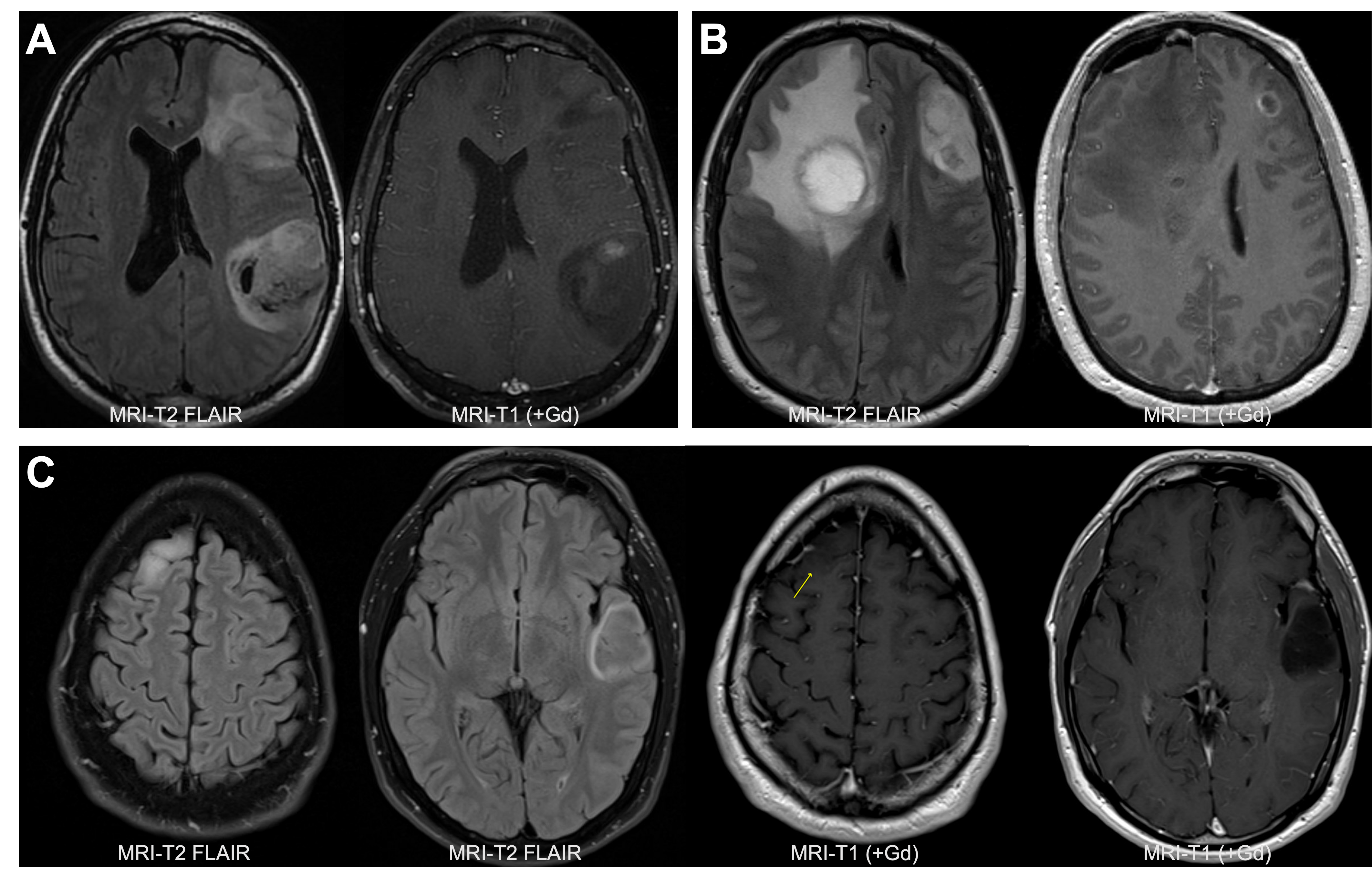


**Supplemental Figure 2:**Pre-operative MRI studies of *IDH*mutant MCG with discordant pathologies in (A) left frontal grade 3 oligodendroglioma and left temporoparietal grade 3 astrocytoma, (B) right frontal pilocytic astrocytoma and left frontal grade 3 oligodendroglioma, and (C) left temporal grade 3 and right frontal grade 2 astrocytoma. For each case, axial T2-FLAIR (left) and T1-weighted post-gadolinium contrast (right) images are shown.

**Supplementary Figure 3:**Time to metachronous versus imaging follow-up time in patients who acquired mMCG in (A) *IDH*wild-type mMCG (p=0.002) and (B) *IDH*mutant mMCG (p=0.003).
